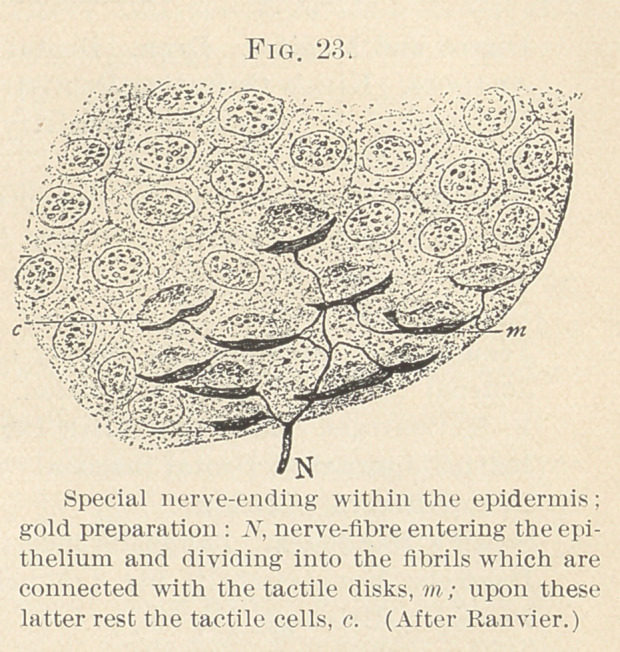# Résumé of the Histology of the Dental Pulp

**Published:** 1902-10

**Authors:** V. A. Latham


### RÉSUMÉ OF THE HISTOLOGY OF THE DENTAL PULP.[1]

BY V. A. LATHAM, M.D., D.D.S., F.R.M.S.

THE consideration of the details of development and structure from a new point of view, I regret to say, is too incomplete, and my opportunities have been too few, to allow of the experimental work being completed in time for this meeting. I can only bring to your notice a number of what seem to me to be important questions bearing upon this subject for your discussion.

When we consider the literature at our command, we feel inclined to ask, Is this reliable? Can we prove these statements? And when we commence our line of proof by experiments, we meet with many obstacles. First, we find our education as regards our microscopic work has been neglected in college, or only given us from one side,—the medical, with no dental bearing at all. What student is taught the principles of optics, illumination, and the use of the condenser, which are so important in this work, when we must differentiate and explain what we see, so as not to give false interpretations? It is often said, "We don't need any such work; we shall not ever use it." And very little interest is shown by the student, because such ideas are presented. Take away the microscope from our profession, and we take away almost all the greatest work, as, for example, the studies of Boll, Sir J. Tomes, Charles Tomes, Legros, Magitot, Leber, Rottenstein, Kölliker, Waldeyer, Von Ebner, Retzius, Mummery, Miller, Miles and Underwood, Michaels, Choquet, Vignal, Gallippe, Huxley, Klein, Charteris White, Paul, Goodsir, Caush, Williams, Goodby, Hopewell Smith, Andrews, G. V. Black, Schaefer, Spec, Sudduth, Bödecker, Broomell, Paris, Arkövy, Vincentini, Wedl, Weil, Morgenstern, Walkoff, Römer, Neumann, Röse, Raschkow.

Only recently Dr. Walkoff has disclaimed Williams's idea of the cement substance being present in enamel, and says that the rods are actually in contact. Why is this? Because of the difference due to micro-training, and the correct interpretation of images.

---

[1] Read before the American Medical Association, Section on Stomatology, at Saratoga Springs, June, 1902.

Dr. Walkoff says the cement-substance theory is due to inaccurate focussing and to the use of objectives having too low a numerical aperture. Hence we see the reasons why our older men studied optics, illumination, and diatom structure, requiring the highest acme of skill, not only to produce the picture, but to interpret the same. One observer may describe hexagonals, another dots, a third clear space and lines,—all in the same field. Who is right?

It is a matter of some pleasure to note that Dr. J. L. Williams has gone so far as to urge the study of the lower forms of life, with a definite object in view. This has been my own desire for many years, and I have always tried to give a few hours to the subject, together with lantern slides and mounted specimens. Our dental students should be taught this subject, and by a teacher who is broad enough to show the close relations of science, medicine, and dentistry. Unfortunately the pressure of manual work is so great that the students seem only too glad to put their microscopic science branches into the smallest limit of time, and they demand the most rapid methods of work; and I am sorry to say it is here the division of sentiments occurs in our professional schools. One set of teachers urge such work as being of little value compared to the operative, prosthetic methods, but they will confine a class of students to a four or six weeks' course on natural tooth forms.

Students classify themselves into many degrees and we are expected to suit them all.

First comes the industrious one, who, if given the scheme and slides, aids himself.

Secondly, the average man, who expects his teachers to give him the cream of all the work ready to digest, and to stand at his elbow all the time.

Again, the slow, dull fellow, who desires constant repetition.

Lastly, the lazy man, who cares for no one's comfort or even his own progress.

There are also difficulties inherent in the subject itself. Take, for example, the study of the physiologic resorption of the deciduous teeth, where the pulp retains its vitality. And here we may ask where is the study of the physiology of our dental structures similar to those given the medical student concerning the action of the stomach, liver and intestines? Physiologic teaching of a nature to call forth any vital interest is at a very low state in the majority of the schools in this country, and it is a detriment to both medical and dental practitioners, as they both work in the field of clinical medicine as well as in the anatomic and surgic.

To return to our pulp. As soon as the so-called absorbent organ reaches the pulp it causes the latter to undergo a change at the point of contact. It is probably during the time required for this change to be effected that the dentine immediately surrounding the tooth-pulp remains safe from dissolution; but once the change sets in, the part of the pulp near by joins in the general attack upon the tooth; *i.e.*, the pulp becomes an absorbent organ, alternately resorbing and laying down new tissue which closely resembles cementum in all details except the lamellar structure of the latter. If the pulp dies the natural process is arrested; roots do not absorb, but remain to obstruct the eruption of the advancing teeth, in some causing alveolar abscess and in others irregularity. This is borne out, as first described by Dr. Theodore Stack, by the faceting of the ends of the roots of the deciduous teeth by the crowns of their permanent successors. Tomes says, "When resorption has progressed so far that only a small part of the crown remains, the enamel is likewise involved, and resorption indentations make their appearance in it also." He has seen, both in animals and in men, many pulps taking on a diseased action, sometimes on account of caries approaching, and sometimes the pulp eating away the walls of its own chamber. Then after a time that process of destruction is stopped and the giant-cells of absorption themselves are calcified.

Another question: How near to the pulp do the micro-organisms in dentine penetrate? So far that more than eighty per cent. are sacrificed. Almost all pulpless teeth are infected, and so give abscesses later. Even the smallest superficial cavity shows the deep penetration and the symptoms of pulp irritation. The extreme difficulty of removing the ultimate fragment of a pulp seems to me worth noting, from a histologic, pathologic, and bacteriologic point of view. That it is difficult is borne out by attempting to pull out a pulp from a tooth that has been cracked open; at the apex of the root the pulp will be found especially adherent. Are these attachments nervous, vascular, or connective tissue? This point has a practical bearing in operative dentistry. In reading papers on treatment of pulps, one sees the statement, "Of the difficulty in removing the colloid material at the very apex when trying to empty cavities, and the difficulty to find any agent that will do it." What this is I am not prepared to say, and I think some pathologic work might inform us of the nature of this material.

If an organism is bathed in its own juice, it ceases to live. The cultivations made at the present time to produce the various serums for dealing with diseases of various kinds are, I believe, based upon that fact. In *dead* teeth a simple extrusion from the alveolus is seen; in *living* teeth, a growth of the alveolar ridge, which is dependent on the live pulp. The wasting of the ridge after removal of teeth is due to *extraction of the dental pulp,* as well as to the local damage caused by removal of the tooth itself; indeed, it may be fairly maintained that the results of the tooth extraction scarcely equal those due to the loss of functional activity of the pulp; for the pulp influences in a marked degree the nutrition of the alveolus and its process,—a *trophic function.* The loss of the pulp may cause a disturbance of the vasomotor equilibrium, in the direction of a paralysis of the vasoconstrictor mechanism, similar to that seen in the familiar experiment upon the rabbit's ear, when section of the sympathetic causes vascular dilatation. The peridental membrane is congested, owing to a loss of resiliency in its blood-vessels,—a condition very likely to cause stasis and so account for the change that takes place in the surrounding alveolar process. If the pulps are removed from deciduous teeth, strictly speaking, resorption does not take place, but, like the change affecting dead permanent teeth, the process is *pathologic,* not physiologic, and is much more slowly accomplished than normally. This is one of the reasons why we see the roots of dead temporary teeth coming through the alveolar margins and causing ulceration of the cheeks and lips. (See Tomes's "Dental Anatomy," page 129.) Gingivitis and alveolitis may be a sequel to degeneration of the tooth-pulps and the sensitiveness of the teeth on the periphery in pyorrhœa alveolaris, which we can compare to the corneal reflex. Hence we must get our microworkers to demonstrate direct nerve connection between the peridental membrane and the so-called odontoblasts. We should have credited these with a vasomotor function, for, situated as they are at the very periphery of the pulp, closely joining the dentine, it can hardly be doubted they must be peculiarly susceptible to even minute variations in the blood-pressure. We must prove the odontoblast cells part of the sympathetic nervous system. Cases have been recorded where an exposed pulp struck by the forceps in extracting, or by a probe, has caused a tonic cramp of the flexor muscles of all the fingers of both hands; in one case the left hand was closed so tightly the patient could not open it and the flexibility of the arms was affected.

Another reason why the pulp should be more thoroughly studied lies in the fact that it is so often diseased. Kay, Magitot, and others report an examination of more than ten thousand teeth; in these, one thousand showed that 18.1 per cent. were affected with pulpitis.

Again, we sometimes forget the strain the pulp is put to; and we must remember that though the teeth are the chief mechanical agents in digestion, they also subserve other functions,—namely:

(*a*) Locomotion (walrus).

(*b*) Speech (man).

(*c*) Digging for food (indirectly aiding digestion).

(*d*) Combat (narwhal, wild boar, tiger).

(*e*) Anchorage (dinotherium).

(*f*) Transportation of things useful to the animal (beaver, elephant).

Thus our comparative anatomy must also be looked into to show the varieties of pulps, their location and function, before we can accept our present views. Professor Röse has been doing excellent work in this line.

In considering the structure of the pulp it is almost impossible to avoid discussing the dentine and enamel, as they are closely united in function and type. Huxley says, "Neither the capsule nor the enamel-organ takes any share in the development of the dental tissues, all three of them—viz., enamel, dentine, and cement—being formed beneath the membrana præformativa, or basement membrane of the pulps."

We are told the epiblast forms the mucous membrane of the body, and in conjunction with it the mesoblast, also the foundation of the pulps, but the true mucous membrane (mesoblast) does not develop till the forty-fifth day of gestation, and is in a stage of evolution, whilst the epiblast cells are completely developed. All cells start alike, but whence this specialization few, if any, have determined. Teeth are, from a morphologic point, the most interesting structures in the oral cavity. Their development in man and mammals is neither simple nor easily intelligible; in the lower animals we find it the simplest. The teeth are originally nothing else than ossified papillæ of the skin and the mucous membrane.

(Compare the development of Selachean's teeth; here we see a simple development of the dental pulp and enamel germ.) The earliest activity is seen about the sixth week of fœtal life,—different periods in different animals. The enamel-pulp, or stratum intermedium, is seen at the eighth week, but most highly developed about the fifth or sixth month, then diminishes in size up to the time of birth. The dentine bulb appears about the sixth week and becomes conical about the ninth week, when the dental sac of the future follicle shows. Calcification of dentine begins about the sixteenth week of fœtal life. About the eighteenth week the sacs of the primitive dental follicles are closed. The salivary glands are found in the second month. First appears the submaxillary gland, in the human at the sixth week. (Chievitz.) Then the parotid, at the eighth week. Finally the sublingual. (Hertwig.)

We all know the epithelial cord (Fig. 1) is epiblastic, and dipping down from the lowest point processes or buds push out. Their growth seems to stimulate the tissue beneath to form little islands which the epithelium envelops, similar to the index finger being forced into a soft ball, and so giving us the illustration known as "the Florence flask stage" (Fig. 2), the under part of the flask being occupied by the subepithelial mass. The flask is also full of epithelium, columnar in variety. The little mass isolated everywhere but at its base is known as the "dentine germ," and sometimes, because of its shape given by its isolation, the dentinal papilla and dentine bulb and its substance is gradually transformed by a series of changes into dentine, enamel, and pulp-tissue. All structures are chiefly composed of cells, but those cells on the periphery of each, next to the basement membrane, are especially to be noted, being elongated, well-marked, and columnar. The pulp germs (dentine germs) are also cells; the outermost just beneath the basement membrane are also specially formed. In or around them (opinions differ as to which) the substance called dentine is formed, afterwards calcified. The cells immediately underlying them are intermediary, and possibly reinforce them when their energy is spent. One question seems to come here: Why should the pulp-organ not receive its own designation rather than that of the dentine? Is the dentine more important, or is it merely due to the fact that workers were surprised at the specially large peripheral cells, and undertook to study these more minutely and so found the origin

Fig. 1.

Developing tooth-pulp. S. reticulum and early calcification.

Fig. 2.

Flask-stage of formative papilla. Invagination of enamel-organ.

FIG. 3.

L. S. pulp. Blood-vessels and nerves.

FIG. 4.

T. S. pulp. Nerve-fibres and blood-vessels.

Fig. 5.

L. S. pulp. Odontoblasts and blood-vessels.

Fig. 6.

Vascularity of enamel-organ and pulp, etc.

Fig. 7.

Vasomotor nerve-supply of pulp. (Special stain.)

of the dentine? It seems a mistake to have so named it, for its *special* significance and function is lost and we had better still regard the island as a "formative papilla" till the various individual structures are well marked, and then continue its name of pulp if still so desired. This name is a questionable one, and indicates more of an embryonal stage than a highly specialized organ. Legros and Magitot state: "From the external surface of the dermis (mucous membrane) arises the dentine bulb or dentinal papilla. Some authors state that the papilla arises in the submucous tissue, but to me it is evidently from the dermis" (page 26). I believe it was the late Dr. M. S. Dean who advocated before the American Dental Association in 1878 that the dentinal papilla is induced. If this be true the origin of a supernumerary dentine papilla is readily accounted for. This follows, and accepts Dr. Magitot's theory, "*That the enamel-organ determines the form and character of the future tooth.* Dursy according to Waldeyer speaks of the semilunar area of tissue extending along each half of the jaw and from which the dentine germs are developed. They project against the enamel-germs while the remainder atrophies. The two horns of the semilunar mass extend from the base of the papilla some distance up and embrace the enamel-organ and dentine germ."

Dr. Andrews, in Kirk's "American Text-book of Operative Dentistry," says, "While the central cells of the enamel-organ are changing, the dentine germ is assuming the form of the future tooth-point," and this seems to be opposed to Magitot's theory. The more work done leads me to accept this, rather than to give the enamel-organ the credit. If we consider the origin, certainly epiblastic cells are first formed, and consequently are older and first in the field, but a section shows we also have the hypo- and mesoblastic cells present; and looking at one of the earliest stages we can obtain, evidence is found that the mesoblastic structures are working as well as the epiblastic, though not quite in so well marked a form. Can the epithelial cord grow without vascular aid? Even if well-defined blood-vessels are not present, we have a secretive or plasmatic osmosis passing through the embryonal cells, and very fine arteries or capillaries (Figs. 3, 4, 5, and 6) in our deeper structures. We are told in Legros and Magitot, the earliest dentine papilla contain vascular canals, but Robin and Magitot both deny the presence of nerve-fibres; if circulatory vessels are present we *must have nerves* to control these vessels, and by careful preparation

I find them in sections, some about the eleventh week. (Fig 6.) Another statement. At no time during the development of the tissues do the dentinal papilla and enamel-organ become united. (Figs. 8 and 10.) Sudduth confirms. Legros and Magitot state that no vessels and nerve-fibres have ever been demonstrated as passing from one to the other. (Fig. 8.) Bödecker disagrees, saying that when we detach the enamel-organ from the dentinal papilla there appears upon the outer surface a delicate fringe which he believes to be the true connection between the enamel-organ and the dentinal papilla. A distinctly marked line is often seen separating the ameloblasts from the odontoblasts, just before the beginning of dentine formation. Later, when the formative cells of the two tissues are separated by a considerable thickness of dentine, we may see a membrane-like structure covering the odontoblasts which appears identical with that bordering the inner ends of the ameloblasts. This seems to suggest that there is an original basement membrane separating the tissues from which these different cells originate, and just before commencing to form the dentine it separates and remains as a covering for both amelo- and odontoblasts. (Williams, J. L., *Dental Cosmos,* 1896, page 113.)

This statement should be used with a microscopic examination, and here we must outline our layers of tissues. If we consider the bulb as a formative organ,—the pulp,—add our specialized cells, the odontoblasts, whose character is that of a new histologic chemic structure with a set function,—*i.e.,* formation of dentine,—to these cells and their membrane, ought we only to apply the term dentine germ or organ when it is a complex structure with complex functions? We have created a special title for the ameloblasts whose analogue is the odontoblast, and in the term enamel-organ we must not forget we include other tissues,—namely:

(*a*) External epithelium (Nasmyth's membrane, Waldeyer).
(*b*) Stellate reticulum.
(*c*) Stratum intermedium (enamel pulp).
(*d*) Basement Membrane.
(*e*) Ameloblasts (internal epithelium).

} Enamel-organ.

If we use this term, we must understand we mean all the layers. The cells of the stellate reticulum are a modified form of those composing the middle layer of the oral epithelium. (Sudduth, Wil-

FIG. 8.

Dentine and enamel-organ joined.

FIG. 9.

Shows dental follicle, or sacculus, and dentine and enamel layers united.

Fig. 10.

Pulp, dentine, and enamel, with Tomes's fibres and stratum reticulum. × 1000.

liams.) The enamel-organ is vascular. (Beale, Howes, Poulton, Williams; but Wedl, Magitot, and Legros deny it.)

To go back, what did these authors mean in the term dentine papilla and enamel-organ, and by stating they were not united? These show they are united (Figs. 8 and 9); but if we mean the ameloblasts are not united to the true bulb, we may find it yet unproved unless we agree that the odontoblast fibres run through the formed dentine and unite with the Tomes fibres (Fig. 10.) Again I find that we have the tissues in contact, but only in certain stages of development, and a variation occurs in studying different animals.

The formative organ consists of many mesoblastic cells, and is analogous to the enamel-organ, so called. At a very early stage (six weeks) we can notice under the epithelial cord a faint aggregation of cells which appear deeply stained, showing active proliferations like a spot of round-celled infiltration in an inflammatory area. Gradually a stroma-like outline appears, as if it were going to wrap around the epithelial cord, with a dark area at the centre of the base which soon becomes semilunar in shape. (Fig. 2.) Now the lateral areas absorb, whilst the central area rises and builds up a papilla, many densely packed cells, so much so that they cannot assume any definite shape as do the cells of the stellate reticulum, yet leaving below room for the stroma of connective tissue, and a special fibrous matrix of delicate, wavy fibres derived from the cells, as seen *only* if the staining has been especially done, vessels which even pass among the odontoblasts and nerves or plexus. This compares with the enamel-organ where we have nothing but cells and intercellular substance. This difference, of course, we can foresee when we consider the origin of the two organs. The one is epithelial, a mere dipping down of the epidermis; the other, derived from mesoblast or subepithelial (true dermal) layer, and so shares its structural changes and marked functions. An apt illustration may make this clear. We have two armies contending for victory standing face to face. The front rank of each army—the enamel-organ—composed of its best,—the special cells, ameloblasts. The other, the fine large odontoblasts; behind these respectively stand ready to step into place when needed the cells of the stratum intermedium and the layer behind the formative odontoblasts, while the centre of each host is formed of reserves. (Figs. 11 and 21.)

Now, suppose each army is contesting the ground, not by fighting, but by tactics. Earthworks are thrown up, and as each side increases the thickness of his fortifications, so the distance between the combatants increases until the ameloblastic army has used up all its reserves, and being cut off from supplies, the remnant perish, leaving nothing but their outwork standing.

During these manœuvres the dentine army, pleased with a substantial fort (throughout which every care was used to keep their complete system of telegraphic operations), in no trouble for a base of supplies and reinforcements, at last stands at ease.

Their front rank men are tried veterans now, stiffened by disuse as the years go on, nearly forgetting their building powers once so quickly done. Caries may try to steal in and destroy their ramparts; then must they begin to wake up and repair, but slowly, clumsily at first, better as they progress to form secondary dentine and so protect and strengthen the point of attack. If not alarmed by the sentry early enough a breach is effected, the enemy (bacterial) pours in. But here we enter a new field of devastation and despair,—pathology.

The odontoblasts, the true dentinal germ, and collectively making perhaps the membrana eboris of some authors, vary in shape. The ends near the new dentine are wider than the inner part. Artefacts may and do occur in our manipulation, but we may find cells varying in shape from a banana to a pear, and the old cells are shorter than the active workers. Underwood says, "The cells which commence the work of dentine formation are thought to be smaller than those which complete it, and this Mr. Hopewell Smith suggests as an explanation of the greater caliber of the fibrils at the pulp end." A view held by Mr. Underwood as more acceptable than the more widely received one, that, as the dentine grows older, the fibril becomes converted into sheath, the sheath into matrix; but this belongs to the field of calcification. (Fig. 13.) The origin of dentine formation is hardly belonging here, except as we include the special function of the odontoblasts in with the *formative* papilla under the head of dentine germ. Briefly, as so much is not yet proved and the subject comes under calcification, we all agree that the formation of dentine is due to active cell proliferation. It must be borne in mind that the tissue is formed before it is calcified, and it is easy to show a layer of formed, but as yet uncalcified, dentine, separating the cells from the completed dentine. We note the chief ideas regarding the functions of the odontoblasts.

*First.*—Mr. Tomes's views are generally received. That each

FIG. 11.

Ameloblasts (beaded), dentine pulp, and odontoblasts.

FIG. 12.

Pulp, odontoblasts, ameloblasts, and matrix. × 1000.

Fig. 13.

Dentine odontoblasts, calcoglobulin.

Fig. 14.

Developed tooth with glands of Serres, *in situ*.

odontoblast converts into dentine by calcification of its outer part, the axis cylinder remains soft as the fibril, and between the two is a semicalcified layer or sheath of Neumann. The subsequent fusion of the conjoining cells shows no line of demarcation, a homogeneous matrix, while the fibrils remain connected with the formative cells.

*Second.*—Klein considers odontoblasts form the matrix, the cells below the fibrils which pass between the odontoblasts. This view also requires a fusion of cells.

*Third.*—Magitot regards the homogeneous substance as the seat of formation of the matrix which is calcified around the soft parts, the fibril remaining connected with the cell, just as the contents of the lacunæ and canaliculi of bone remain in the calcified matrix. Here we note that no part of the cells is converted into matrix. For some reasons Magitot's theory seems more reasonable and built on a basis with bone and cementum. If the enamel-organ is the formative builder of the tooth, why does the dentine begin to calcify at the tip or within its substance first, even though the enamel is formed earlier?

The pulp fibres are the origin of the dentinal basis substance. (Purkinje and Raschkow.)

Schwann believes the pulp produces an ossified substance, and calls it dentine.

Baume says, "The odontoblasts secrete a material which calcifies, rather than that they are themselves converted."

Waldeyer says the central remains of the odontoblasts are found as the dentinal fibres.

Morgenstern thinks the pulp transudes a substance which contains salts of lime without odontoblastic aid, which is taken up by them, accumulates, and passes out of the peripheral border.

"The *first layer of cells formed* on the pulp surface beneath the ameloblasts are *not* odontoblasts, but develop into fibres, and on the actual surface these fibres blend together and form a membrane which lies just beneath the ameloblasts." (Paul.)

When dentine is forming, Mr. Mummery (1891) showed the appearance of connective fibres in advance of the line of calcification. In young teeth a reticulum of fibres was seen passing between and enveloping the odontoblasts. They seem to originate from the connective tissue in the formative pulp near the odontoblast layer, and are evidently the foundation of the dentinal matrix, so the lime can be deposited like cartilage matrix to bone.

From a comparative study we must remember the same pulp may make many varieties of dentine, and we will use Mr. Chas. Tomes's table as a reminder.

A. Dentine developed from odontoblasts.

1. Hard dentine, tubes, no vessels, simple pulp (man).
2. Plici-dentine, the same folded, myliobates, labyrinthodon, etc.
3. Vasodentine, tubes, containing vessels and nothing else. (Hake.)

B. Dentine developed from osteoblasts.

1. Osteo-dentine, channels, containing vessels and all other pulp tissues (pike).

The formative or pulp papilla forms and makes the tooth-shape when completed. (Fig. 14.) The enamel calcifies and joins the dentinal organ, this being to the detriment of the pulp-organ, atrophies, which absorbs gradually as the tooth grows older, and the primary eruption stage past, leaving the fully formed but smaller pulp to carry on the future nourishment and sensibility of the tooth.

*The pulp is the foundation of the dentine germ until after calcification is completed.* From then so long as it is vital it is the chief source of nourishment, nervous influence, and safety to the life of the tooth. (Fig. 6.) It is able to prevent the damage, to some extent, of micro-organisms. In its embryonal stage it sustains the odontoblasts in their function. The pulp is the rough miniature of the tooth, but sometimes off-shoots are found running out as far as the surface of the enamel, carrying with them acute sensibility. (See case reported, *University of Michigan Dental Journal,* March, 1902.) The pulp is extremely liable to changes in temperament, disease, thermal conditions, nervous irritability, according to age and the species of animals in which found. The recent (1901) anatomic work of Dr. W. Lepkowski, Cracow, Poland, on the distribution of the vessels in the teeth of man will be a great help.

This monograph is a continuation of his brochure on the vascular distribution in the teeth of mammals (1897), and I cannot do better than quote his words in relation to the blood supply. "In an embryo, 7 mo., the inferior alveolar artery gives one branch for each tooth-germ which enters at its base. As it progresses the walls thin, so that they cannot be distinguished from the veins accompanying it. (Fig. 15.)

Fig. 15.

Blood-supply tooth plexus under odontoblasts. (Injected.)

Fig. 16.

Deciduous molar. Permanent bicuspid (injected). Periosteal vessels.

Fig. 17.

Raschkow and Boll nerve-plexus in pulp. Medullated nerve-bundles. (Special stain).

The vessels rise to the apex of the pulp and there spread out fan-like from the point to the base of the tooth-germ as capillaries. They proceed *between* the odontoblasts *up to* the *dentine* layer, and there form broad loops which unite to each other. At the base of the tooth the vascular net is always denser and more interwoven than towards its point. This arrangement of vessels follows the arrangement of odontoblasts. With carmine-stained specimens a broad seam of odontoblasts at the base of the tooth-germ just where the vessels also are present in greater density; towards the apex of the tooth-germ, the breadth of the odontoblast seam decreases, and then the net-work of the vessels becomes more loose. This is easily explained, for it is on the base that new substance is deposited for the tooth and the vessels and odontoblasts are chiefly concerned in the process. The vessels which externally surround the enamel-organ are connected with the pulp-vessels. (Fig. 6.) The vessels originate in the interalveolar arteries which supply the spongy bone of the maxillæ. They spread out in dense weave at the surface of the enamel-organ, but do not, however, penetrate between the cylinder cells which line the enamel-organ and play an active part in the formation of the enamel; where more enamel exists, the net-work of vessels is denser. (Fig. 16.) When the activity of the enamel cells ceases, the vessels also slowly undergo a retroformation. Within the tooth the formative activity of odontoblasts and vessels continues until the dentine of the crown and roots is built up. The disappearance of the vessels on the enamel-organ begins at the summit of the tooth, and proceeds in the direction of the root. Of the pulp-vessels, individual vessels or also bundles of them occasionally separate, perforate in places the dentine layer and the enamel layer, and obtain connection with the vessels surrounding the tooth-germ on the outside. In dogs the enamel thickness is greater than in human teeth, hence they have a richer vascular system.

When do the odontoblasts first appear, and if the odontoblasts are nerve-cells they must be of epiblastic origin, are questions I have not yet decided.

So far the vascular supply of the odontoblasts seems fairly well proved to be a plexus, and with it comes up the nerve-plexus of Raschkow, and Boll stated that the fibrils inosculate with the branches of the odontoblasts. (Fig. 17.) Clinical phenomena favor such a conclusion. Thermal changes can be perceived long before the plexus or pulp is exposed; and why should such stimuli as dilute acids or slight touches of a steel instrument be felt if no path between the soft tissues of the dentinal and nervous system existed? The thermal changes might pass, but hardly the effects of a dilute agent, as silver nitrate or zinc chloride, without a nervous system of communication.

Another disputed point is whether or not besides the nerve-endings in the pulp there were also nerve-endings in the solid portion of the tooth. While some believe the nerve fibrils end only *among* the bodies of the odontoblasts, at the periphery of the pulp, others think that they may *actually penetrate the dentine* itself, inasmuch as it is known that if the gum be retracted the dentine is sensitive at the margin of the enamel. Some ask the question, "Do the nerves of the dental pulp originate in the odontoblasts?" We say no, they enter from the apical foramen and pass upward as medullated, then lose their sheath and go as nerve-fibrils to the odontoblasts as a plexus. (Fig. 17.) If odontoblasts are secretive, may they not be hypoblastic, and so explain their columnar shape and function as well as forming the mesoblastic layer, which is really the connective-tissue matrix, but has a *characteristic point that in this tissue the fibrils never join to form connective-tissue fibres.* We may have nerves ending in connection with these epithelial cells, just as we do in the gustatory cells, olfactory cells, etc. I do not remember seeing any particular statement or work stating why, or even demonstrating the reason why, we have placed the odontoblast cells as mesoblastic. If we decide odontoblasts (membrana eboris) to be dentine builders, secretory cells, cannot they resemble the special epithelium products as do the secreting glands (buccal, pancreas), which are originally developed from a layer of epithelium, both epi- and hypoblastic, by an invagination or downward growth of cells covering in the consolidating (mesoblastic) connective-tissue formative cells? The apex is the first differentiating point, with a gradual descent on either side of the conical bulb till it reaches the neighborhood of the cement region, the odontoblasts ending here in many slides at this stage. Compare the glands of Lieberkühn in the intestine, simple tubular, and those of frog's skin, simple sacs. The differences are for the most part modifications of arrangement, with a view to increasing the secreting surface at the cost of as little space as possible. Whatever degree of complexity in a secreting area, its essential points remain the same. A layer of secreting cells (epithelial) usually on a basement membrane, surrounded by capillary blood-vessels, supported in connective tissue, bathed by lymph passing through the spaces in the matrix—cells varying in shape according to their location, pressure, and function. We must remember we have two distinct layers of epithelial cells in the epithelial band,—the lowest, the stratum Malpighii, being polygonal columnar, and forms the germinal layer, a possible combination of hypoblastic epiblast, and again in some specimens joining the mesoblast. Here is room for investigation if the epiblast or hypoblast may not be the origin of the odontoblasts. We know the cœlom theory of Hertwig has received many careful supporters in the light of present researches. A careful study of slides will show in some animals a marked differentiation into two layers of the inner tunic of the enamel-organ nearest the formative pulp surface, in some places united, in other places they have been separated, one following the enamel-organ, the other remaining on the apex of the pulp. In further stages a deposition of homogeneous material is being laid down, on this line between the two layers and at a different rate of growth. *The most important point seems to me to prove the origin of these cells,* to study their function; because if pure secreting epithelium, if odontoblasts are of connective-tissue origin as described by Piersol and others, if sensory nerve-organs entering the tubules, it is evident we can hardly have described their function correctly. It may be asked, is the odontoblast of connective-tissue origin—morphologically identical with nerve-cells and axis cylinder fibres (Boll, Morgenstern, Römer), or analogues to bone, differing by being derived from embryonal connective tissue, having fibres passing into the dentinal tubules, similar to the soft uncalcified perforating fibres of Sharpey. (Kölliker.) (Fig. 14.)

When a tissue is found foreign to its position, we call it a pathologic condition; and in this connection I wish to call attention to those specimens of myxomatous pulp whose whole surface is covered with pavement epithelium, of which I have specimens. I believe Dr. Baker,[1] England, also showed a specimen in 1892, at the British Dental Association, which was covered with squamous epithelium, and Römer has lately called attention to this condition. Can these be inclusions originating from the epithelial cord or an overgrowth like a papilloma? Dr. R. T. Stack, 1897, Professors

[1] Journal British Dental Association, 1892, 1897, 1902.

Tomes, Dentz, of Utrecht, and Mummery have called attention to the bulbous dilatations in connection with the dentine tubes. If processes of the odontoblasts are pulled out, and traced to show the ramifications of the tubules and so on to definite endings in the enamel as a bulbous shape in ground sections?

Is it yet proved how far the dentine of a tooth without a pulp may be considered dead? How far nutrition in the sluggish form required by dentine may not be carried on in the collateral plasmic circulation through the cement corpuscles, and these bulbous or fusiform dilatations in the dentinal tubules more especially being found in temporary teeth dentine. In teeth with incompleted roots they are numerous, as well as in those irregularly placed in the mouth and which are imperfectly developed, says Patsch. So far as I have gone in my experiments on special staining I have, I believe, obtained some slides that show the nerve relations and arrangements of the odontoblasts that will be given later. (Fig. 18.) At present the best illustration that I know is under the termination of the sensory nerve-endings. We know the medullated nerve-fibre loses its medullary substance (white matter of Schwann) before reaching its ultimate distribution. They are especially plentiful in the sympathetic system, as well as in the cranial nerves and the olfactory. Whether the fibre be motor or sensory bears no relation to its size; the length seems to directly influence its diameter, since we find long distance fibres are thicker than those only going short distances, and this may account for the very small nerve-fibres in connection with the odontoblasts.

Whether we can liken teeth to the organs of special sense is not yet proved, but I think a brief review of the peculiar modifications of shape and structure which the epithelial cells undergo and form the organs in which the nerves terminate as neuro-epithelium will illustrate my findings in the nerve relations of odontoblasts fairly well. Any one who has done special histologic work in isolating neuro-epithelium from the nose, rods and cones of the retina, hair-cells of Corti's organ, and other parts of the membranous labyrinth, the taste buds, can find another example in the teeth. Briefly, two elements are found, the nuclear and the outer, peripherally directed segment, which is highly specialized and often ends in stiff, rigid, hair-like processes. The outer part receives the external impressions, whilst the inner segment is in close union with the nerve-fibres. Within the Malpighian layer we find the tactile

Fig. 18.

Nerve-supply of pulp and vessels. (Weigert stain.)

Fig. 19.

Terminal nerve-fibrillæ within odontoblastic zone, passing into the intercellular substance of odontoblasts which are out of focus. (Gold method.)

Fig. 20.

Medullated nerves, pulp passing under odontoblasts. (Special stain.)

Fig. 21.

Odontoblasts, fibrillæ, dentine, and enamel.

cells, simple or compound. The former, small, oval, nucleated cells, possibly modified ganglionic. The terminal fibres pass between or on the side of the tactile cell, and are lost in its substance. (Figs. 22 and 23.)

In the pulp we find the nerve-fibres originating from the medullated nerves, which are non-medullated, becoming smaller until the neurilemma disappears and the bundles of new fibrillæ continue as naked axis cylinders, forming a ground plexus; these later fibrillæ break up into their primitive, which, interlacing, form terminal plexuses just beneath the odontoblast layer (Fig. 19), near the line of all bodies or basement membrane, which, so far as I believe, is not demonstrated, but is best known and described as the basal layer of Weil, and as such more easily placed. The nerve-plexus fibrillæ cross this in T. S. developing teeth (cut by the freezing method or Weil's, stained in borax-carmine—gold chloride), and run between the odontoblast cells. (Figs. 20 and 21.) The majority so far being lost sight of, but here and there fibrillæ may be seen passing beyond the periphery of the cells into the forming dentine and specimens show the same, as well as odontoblastic processes. Whether blood-vessels are found stretching beyond the cortical layer of the pulp into the basal layer of the membrana eboris has been denied, as well as even the existence of this layer by von Ebner. It is in the crowns of teeth (fully formed) where the basal layer of Weil is most marked, below the layer of dentine that is first completed, says Mr. Mummery, and his description of the fibres passing from the pulp between the cells to the dentine across Weil's layer, showing vessels in the basal layer, I concur in.

BIBLIOGRAPHY.

Legros and Magitot. Dean. Dental Follicle.
Andrews. Kirk's Operative Dentistry.
Marshall, J. S. Operative Dentistry.
American System of Dentistry.
Broomell. Anatomy and Pathology of the Teeth.
Tomes, J. and C. Dental Anatomy.
Dental Cosmos.
Items of Interest.
Transactions Odontological Society of Great Britain.
Journal British Dental Association.
F. E. Constant. Journal British Dental Association, p. 429, 1899.
British Journal of Dental Science.
Dental Record (London).
Scheff's Handbuch. Zahnheilkunde.
Deutsche Monatssschrift für Zahnheilkunde, etc.
Piersol. Histology, 4th ed., p. 152.

## Figures and Tables

**Fig. 1. f1:**
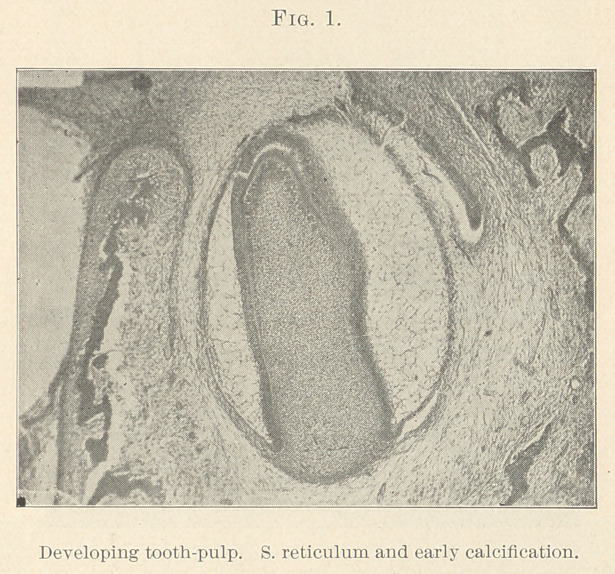


**Fig. 2. f2:**
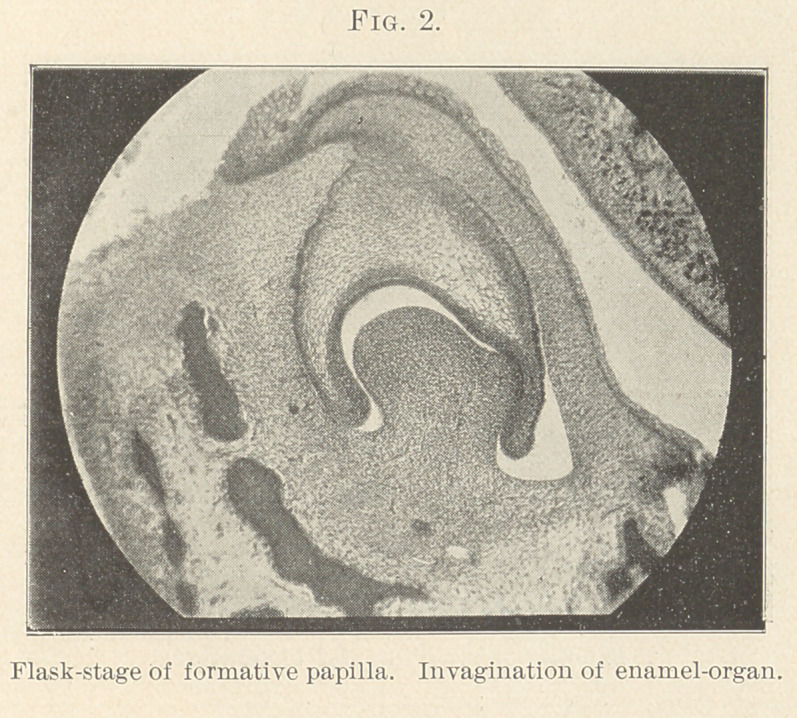


**Fig. 3. f3:**
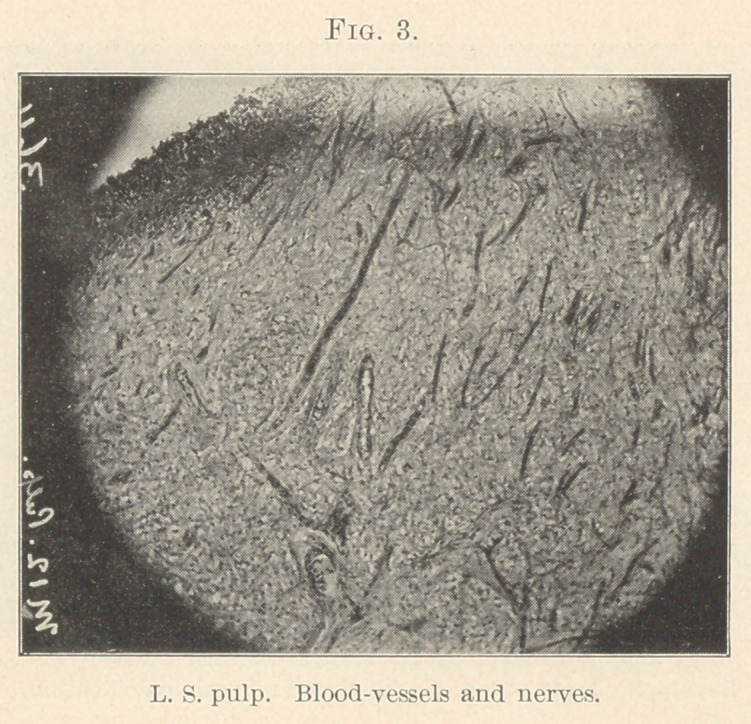


**Fig. 4. f4:**
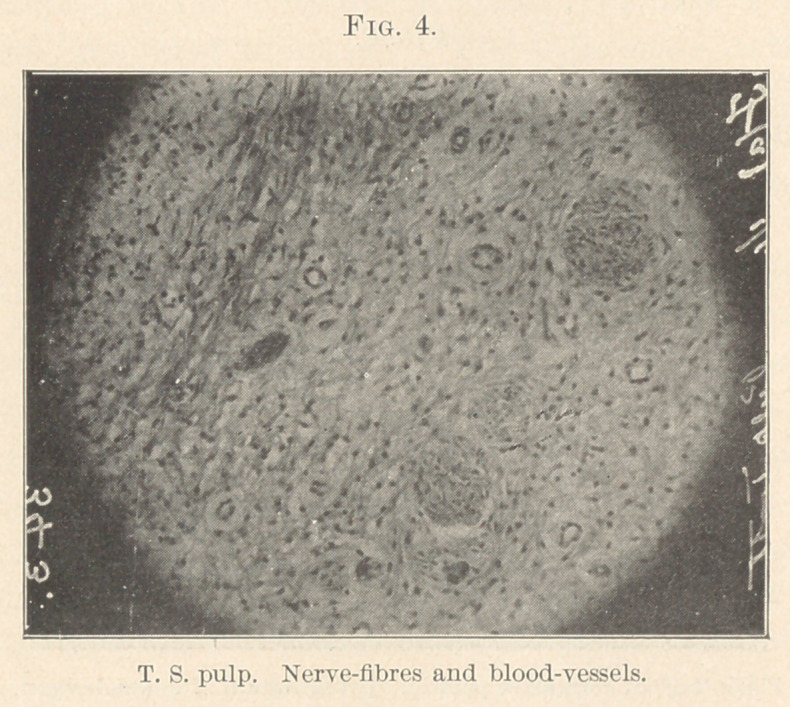


**Fig. 5. f5:**
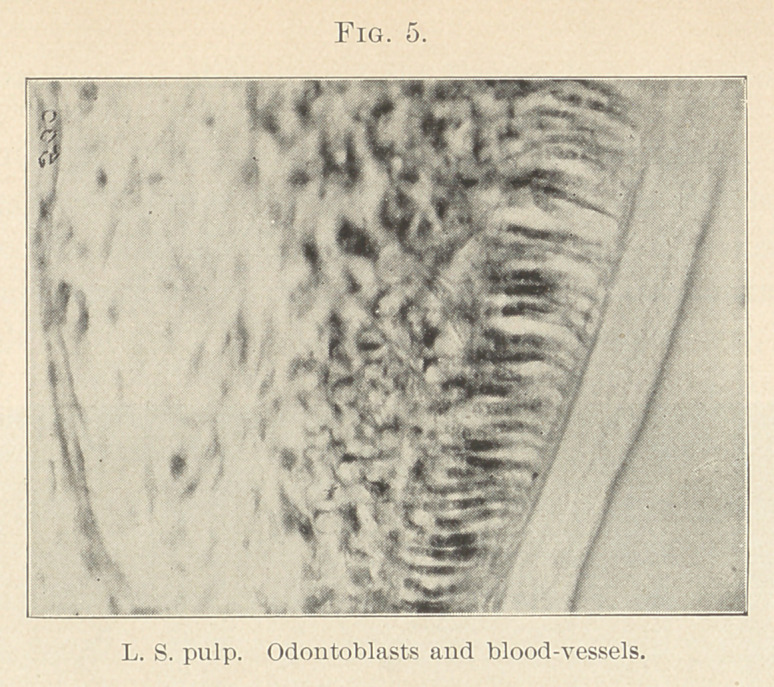


**Fig. 6. f6:**
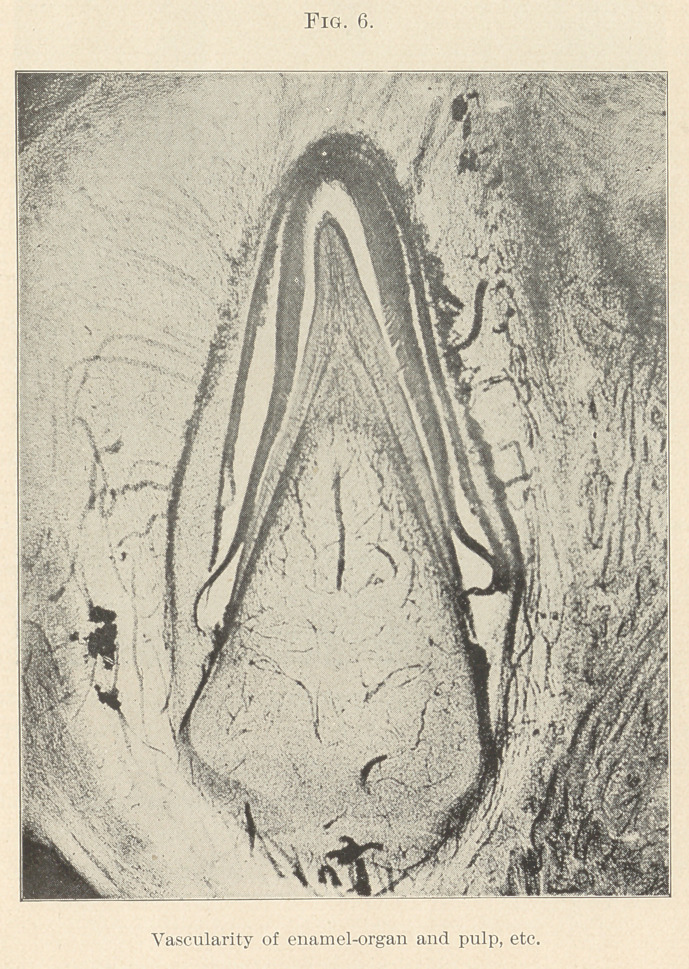


**Fig. 7. f7:**
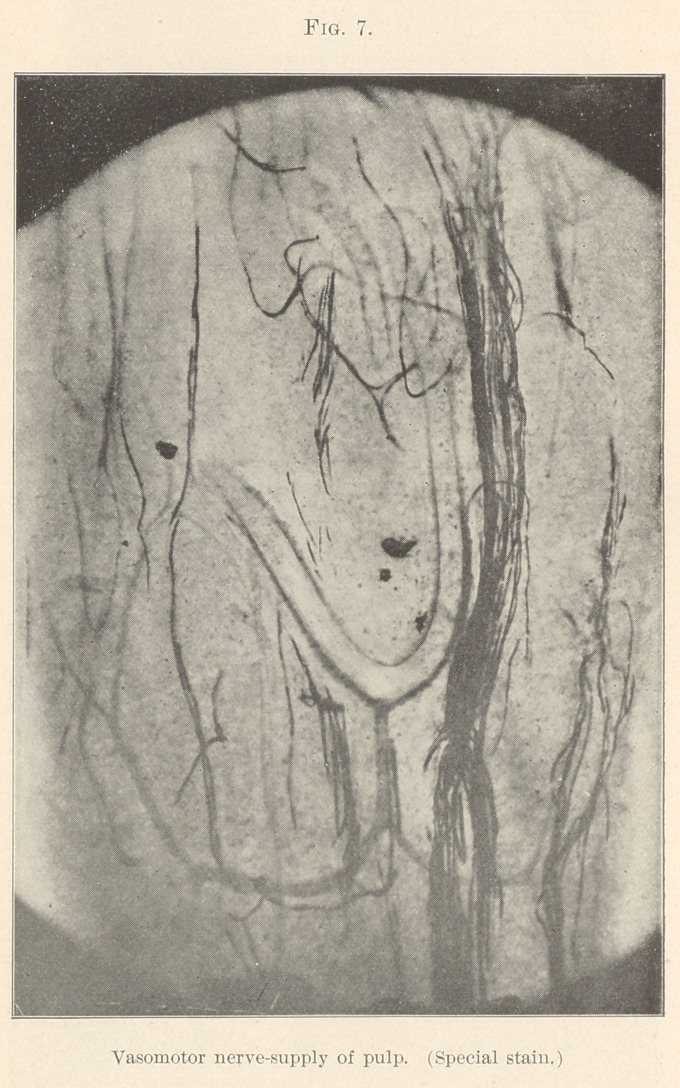


**Fig. 8. f8:**
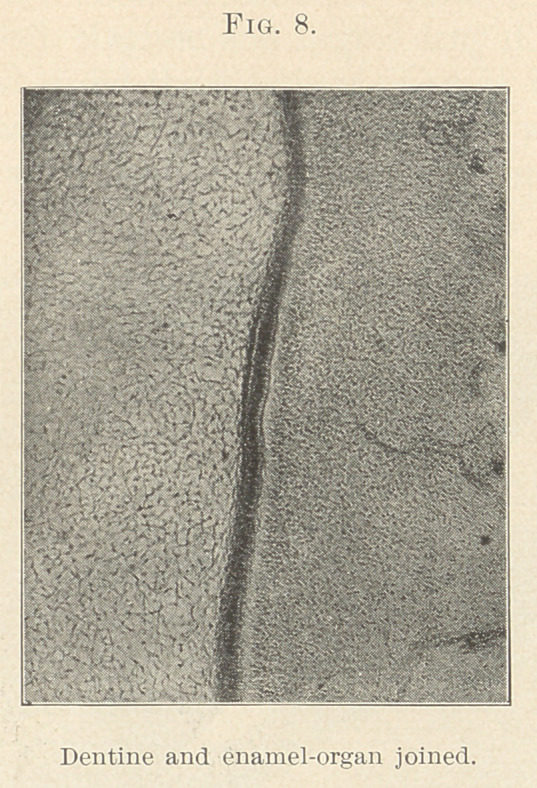


**Fig. 9. f9:**
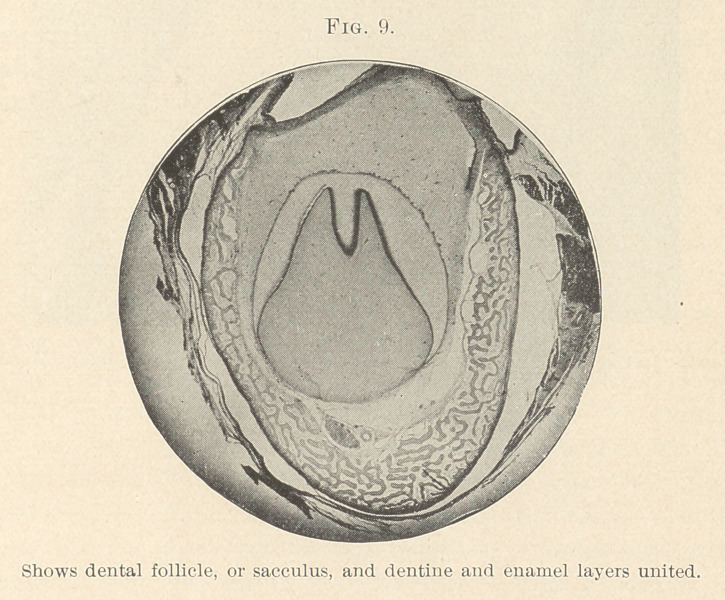


**Fig. 10. f10:**
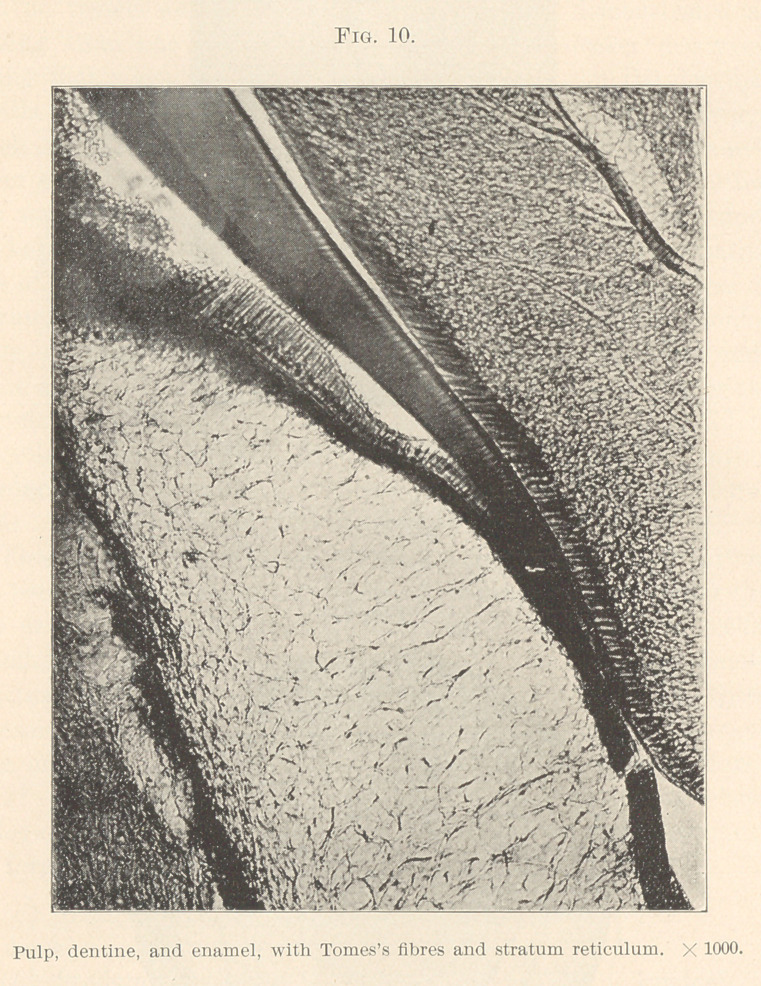


**Fig. 11. f11:**
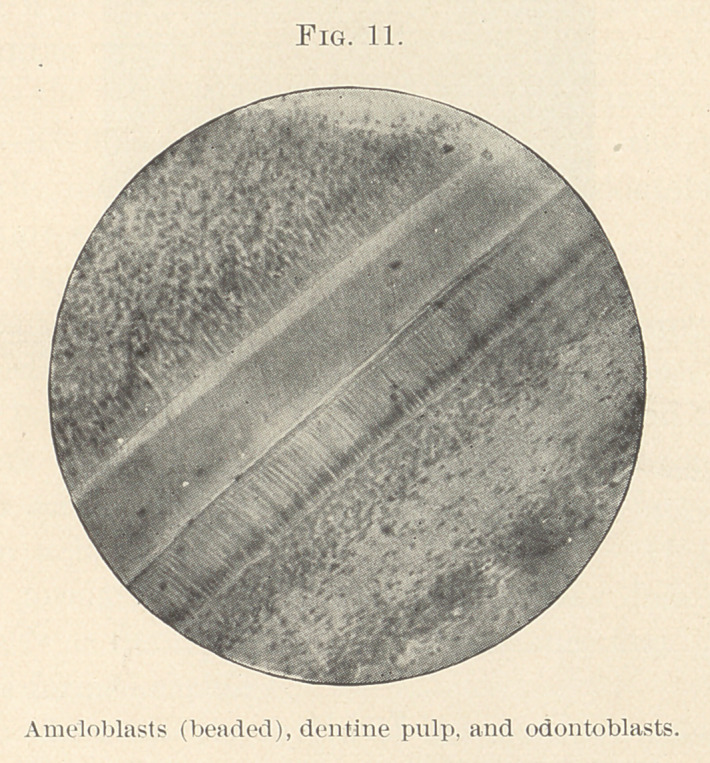


**Fig. 12. f12:**
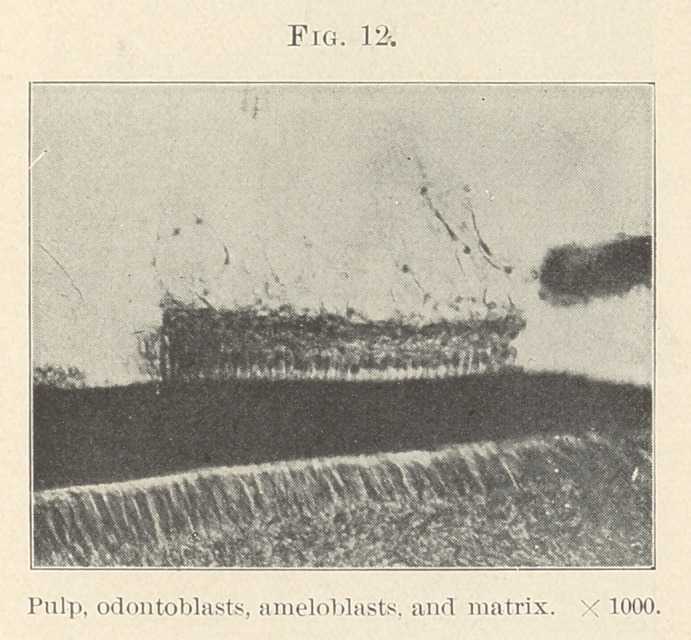


**Fig. 13. f13:**
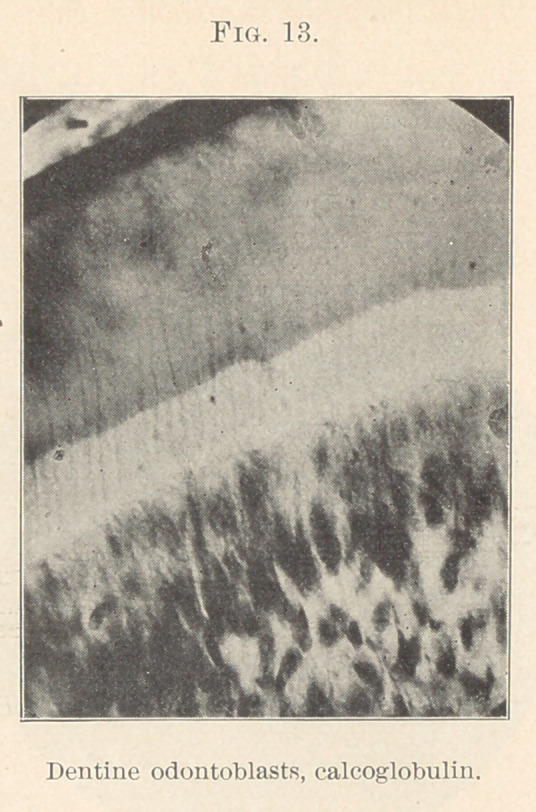


**Fig. 14. f14:**
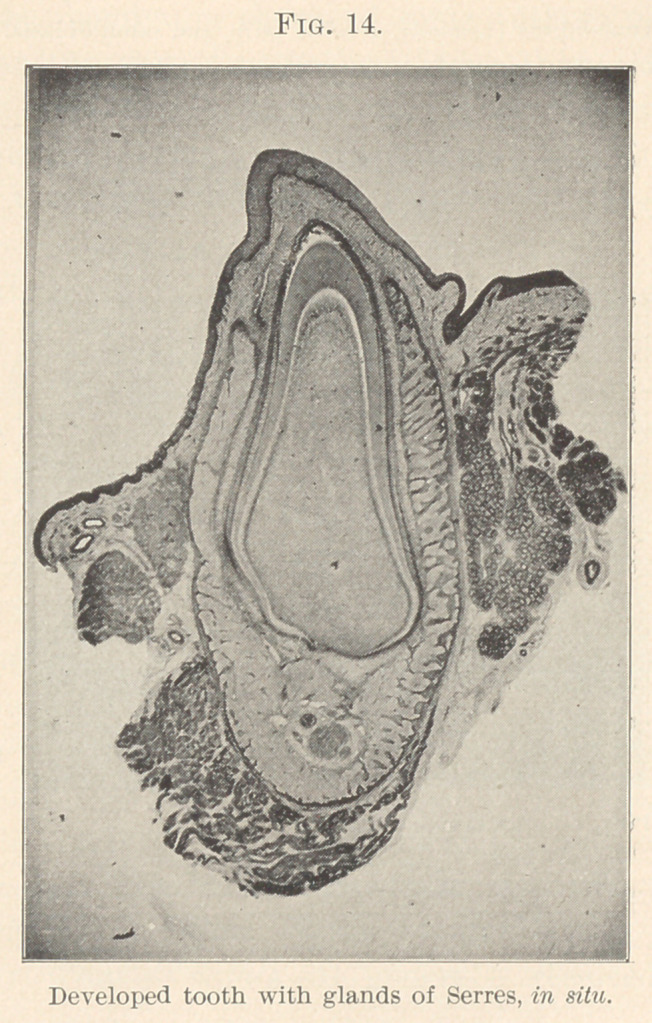


**Fig. 15. f15:**
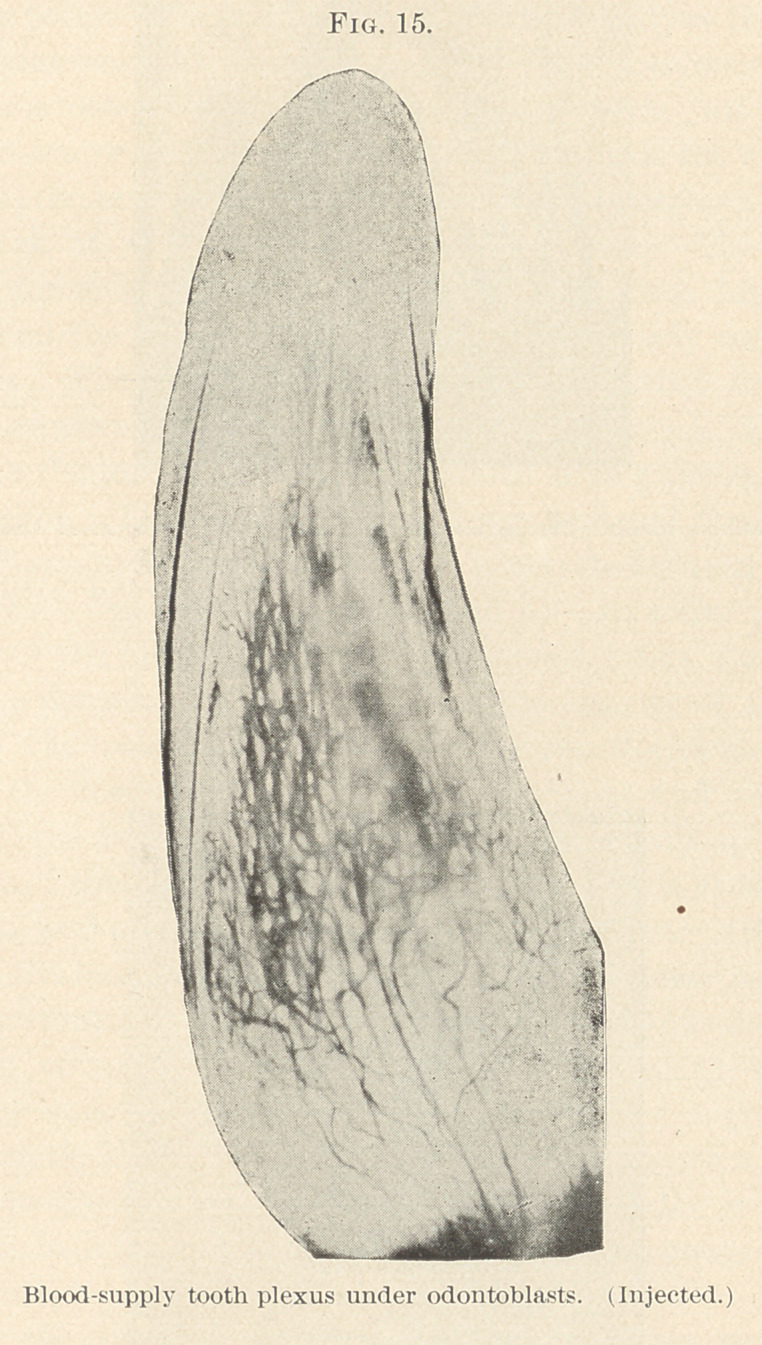


**Fig. 16. f16:**
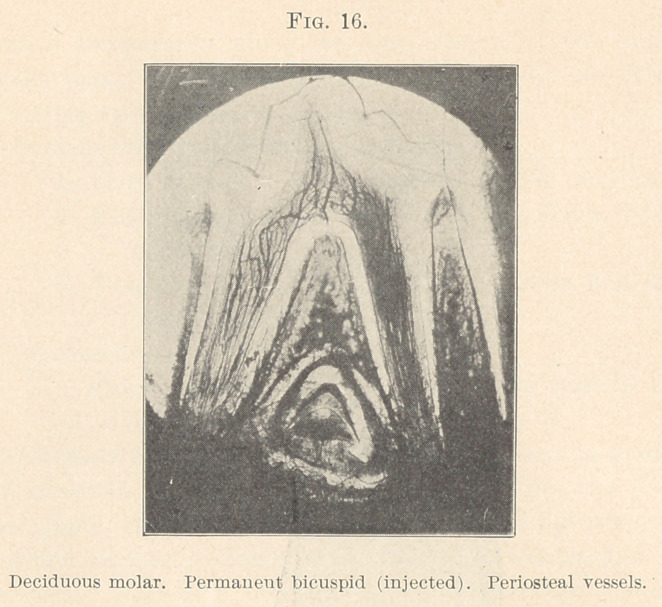


**Fig. 17. f17:**
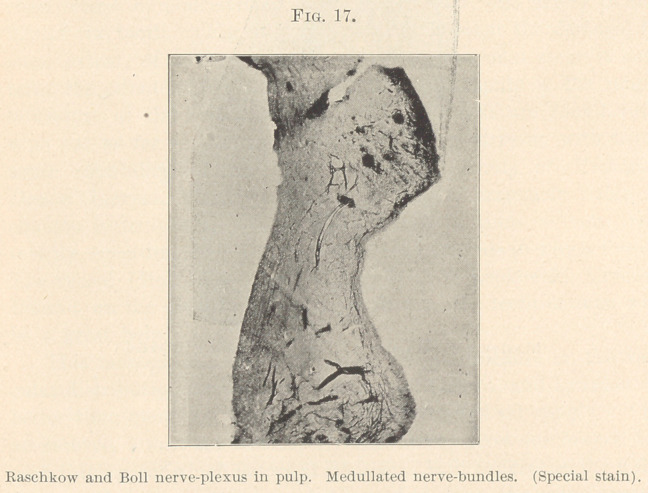


**Fig. 18. f18:**
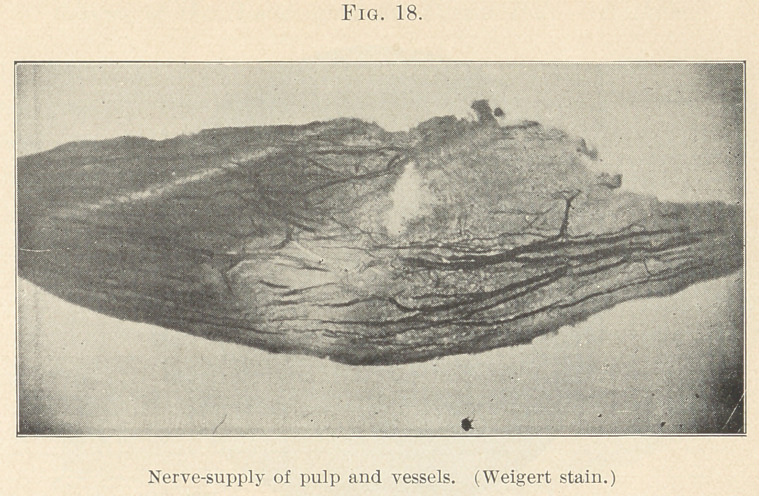


**Fig. 19. f19:**
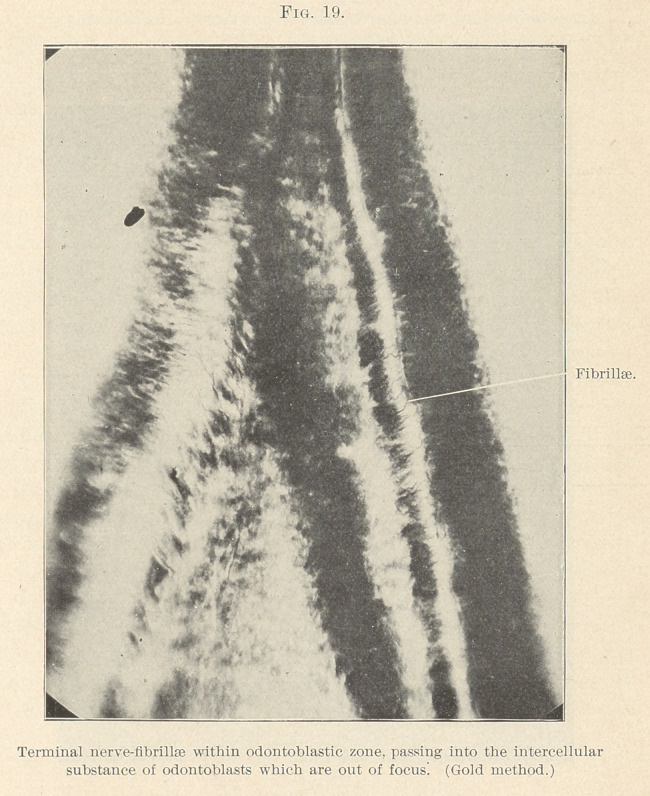


**Fig. 20. f20:**
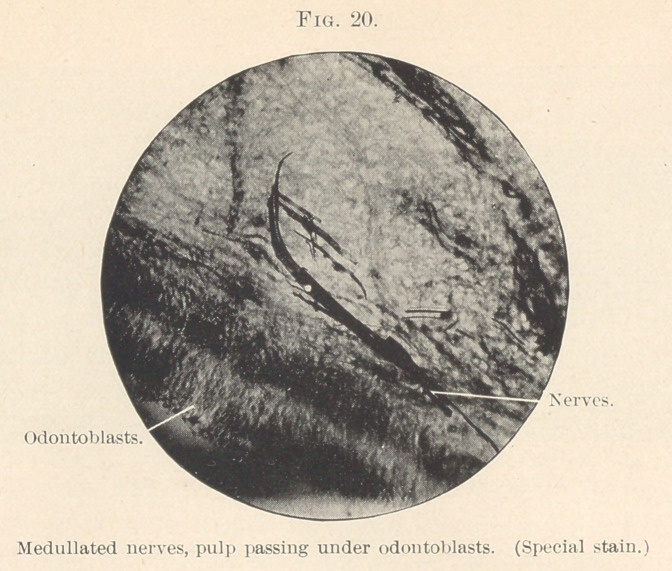


**Fig. 21. f21:**
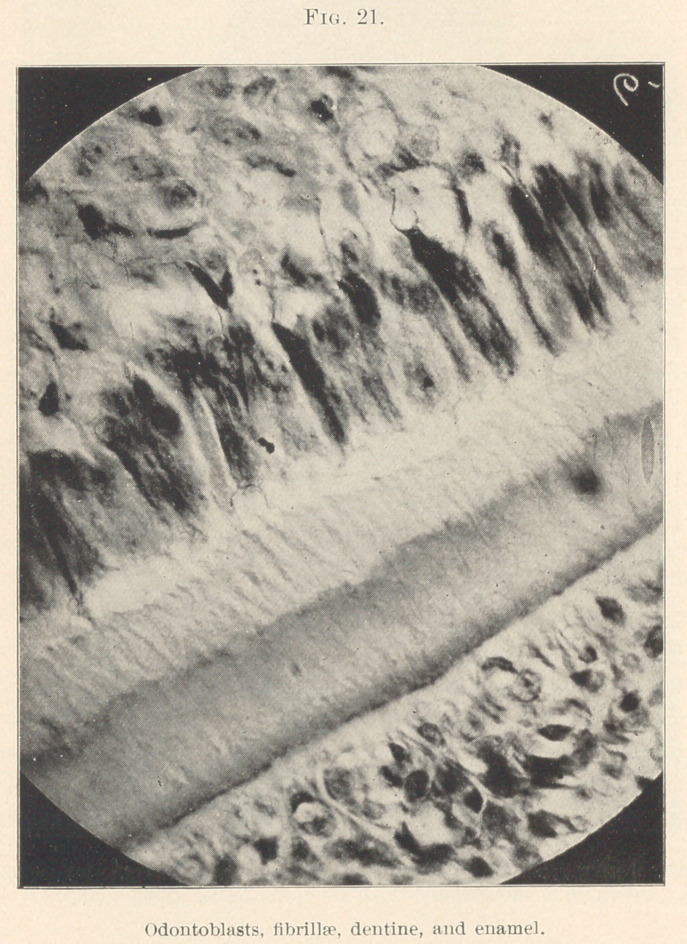


**Fig. 22. f22:**
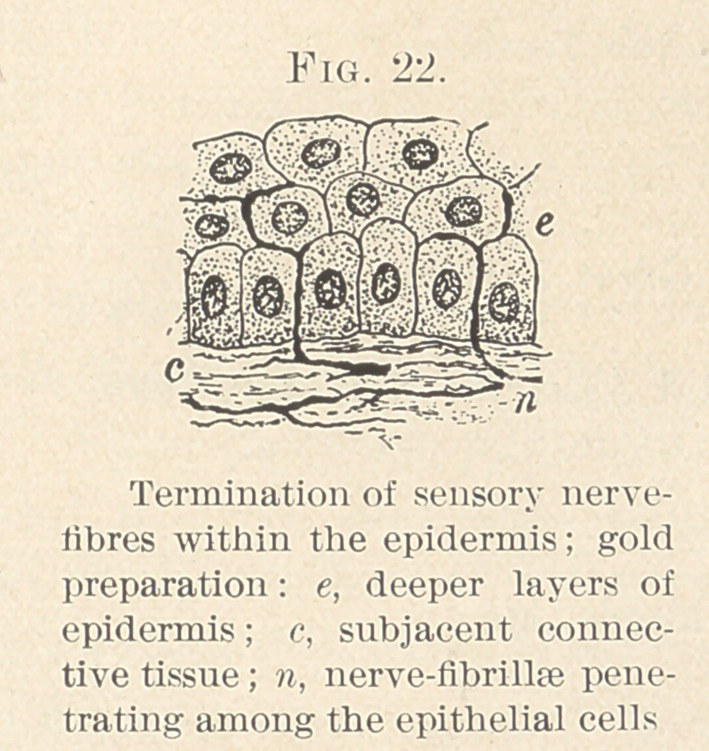


**Fig. 23. f23:**